# The association of circulating systemic inflammation with premature death and the protective role of the Mediterranean diet: a large prospective cohort study of UK biobank

**DOI:** 10.1186/s12889-024-18888-x

**Published:** 2024-05-30

**Authors:** ShiJian Liu, Ruiming Yang, Yingdong Zuo, Conghui Qiao, Wenbo Jiang, Weilun Cheng, Wei Wei, Zijie Liu, Yiding Geng, Ying Dong

**Affiliations:** 1 Department of kidney, the 2nd Affiliated Hospital of Harbin Medical University, Harbin, 150081 China; 2 Department of Nutrition and Food Hygiene, School of Public Health, Key Laboratory of Precision nutrition and health, Ministry of Education, Harbin Medical University, Harbin, 150081 China; 3 Department of General Surgery, The Second Affiliated Hospital of Harbin Medical University, Harbin, 150081 China; 4 Department of Endocrinology and Metabolic Disease, the 2nd Affiliated Hospital of Harbin Medical University, Harbin, 150081 China

**Keywords:** Circulating systemic inflammation, Premature death, Mediterranean diet, Cohort, UK biobank

## Abstract

**Background:**

Although previous studies have identified specific circulating inflammatory markers associated with the risk of mortality, they have often overlooked the broader impact of a comprehensive inflammatory response on health outcomes. This study aims to assess the association between circulating systemic inflammation and age-related hospitalization and premature death, as well as explore the potential mediating effects of various dietary patterns on these associations.

**Methods:**

A total of 448,574 participants enrolled in the UK Biobank study were included. Circulating C-reactive protein(CRP), white blood cell count(WBC), platelet count(Plt), and neutrophil/lymphocyte ratio(NLR) were measured, which were used to establish a weighted systemic inflammatory index of inflammation index(INFLA-Score). Dietary intake information was documented through 24-hour dietary recalls, and dietary pattern scores including Dietary Approaches to Stop Hypertension(DASH), Mediterranean(MED), and Healthy Eating Index-2020(HEI-2020) were calculated. Cox proportional hazards regression models were performed to assess the associations between INFLA-Score and age-related disease hospitalization, cause-specific and all-cause premature death.

**Results:**

During a median follow-up of 12.65 years, 23,784 premature deaths were documented. After adjusting for multiple covariates, higher levels of CRP, WBC, NLR, and INFLA-Score were significantly associated with increased risks of age-related disease hospitalization(HR_CRP_=1.19; 95%:1.17–1.21; HR_WBC_=1.17; 95%:1.15–1.19; HR_NLR_=1.18; 95%:1.16–1.20; HR_INFLA−Score_=1.19; 95%:1.17–1.21) and premature death(HR_CRP_=1.68; 95%:1.61–1.75; HR_WBC_=1.23; 95%:1.18–1.27; HR_NLR_=1.45; 95%:1.40–1.50; HR_INFLA−Score_=1.58; 95%:1.52–1.64). Compared to the lowest INFLA-Score group, the highest INFLA-Score group was associated with increased values of whole-body and organ-specific biological age, and had a shortened life expectancy of 2.96 (95% CI 2.53–3.41) and 4.14 (95% CI 3.75–4.56) years at the age of 60 years in women and men, respectively. Additionally, we observed no significant association of the INFLA-Score with aging-related hospitalization and premature death among participants who were more adhering to the Mediterranean (MED) dietary pattern(HR_Aging−related hospitalization_=1.07; 95%:0.99–1.16;HR_Premature death_=1.19; 95%:0.96–1.47).

**Conclusion:**

A higher INFLA-Score was correlated with an increased risk of age-related hospitalization and premature death. Nevertheless, adherence to a Mediterranean (MED) diet may mitigate these associations.

**Supplementary Information:**

The online version contains supplementary material available at 10.1186/s12889-024-18888-x.

## Introduction

Globally, premature death is becoming a public health challenge, placing a greater burden on socio-economic aspects [1]. Current evidence indicates that older individuals tend to exhibit heightened circulating inflammatory biomarkers with aging. Although numerous studies have examined the association between elevated circulating inflammation biomarkers and various age-related noncommunicable diseases, comorbidities, and all-cause mortality risks [2–7], conflicting results persist. For example, a prospective study involving 15,828 patients with coronary heart disease showed that after multi-variable adjustments, circulating C-reactive protein (CRP) was not independently associated with an increased risk of adverse cardiovascular events, cancer mortality or all-cause mortality [8, 9]. Another study indicated that the neutrophil/lymphocyte ratio (NLR) was an insensitive predictor for atherosclerosis progression and the occurrence of vascular events [10], and white platelet count (Plt) was also not associated with CVD mortality [11].

Traditionally, previous studies have predominantly focused on examining the impact of specific inflammatory biomarker on mortality. However, the intricate interactions within the inflammation-immunity process and their implications for health outcomes remain largely unknown. Given the multidimensional complexity of the inflammatory process, there is an urgent need for a comprehensive index to assess the systemic inflammation state. Recently, a composite blood-based inflammation index (INFLA-Score) has been developed to synthetically assess the systemic inflammation state, incorporating parameters such as CRP, Plt, WBC, and NLR [12, 13]. Although the INFLA-Score has been shown to be positively associated with several age-related mental health conditions [14, 15], limited research has explored its relationship with aging-related health outcomes, particularly in terms of hospitalization, cause-specific mortality, and all-cause premature death.

Additionally, epidemiological research and clinical trials have consistently shown that a healthy dietary pattern, such as the Dietary Approaches to Stop Hypertension (DASH), the Mediterranean diet (MED), and the Healthy Eating Index (HEI), exerts a modifying effect on reducing circulating inflammatory status by improving the circulating content and distribution of inflammatory factors [16–20]. However, it remains unclear whether individuals adhering to a healthy dietary pattern can mitigate systemic inflammatory states and, to some extent, enhance the long-term survival of aging-related health outcomes. Consequently, it is imperative to investigate the modifying effect of a healthy dietary pattern on the association between systemic inflammatory states and aging-related health outcomes. Such exploration could provide valuable insights into dietary management strategies aimed at prolonging life expectancy.

In this study, we investigated the association between the INFLA-Score and various age-related health outcomes, premature death (both all-cause and cause-specific), and life expectancy in a prospective cohort study from the UK biobank. Furthermore, the study also examined the modifying effects of healthy dietary patterns on reducing the risks of aging-related hospitalization and premature death, thereby providing practical guidance for clinical practice.

## Method

### Study population

**Fig. 1:**
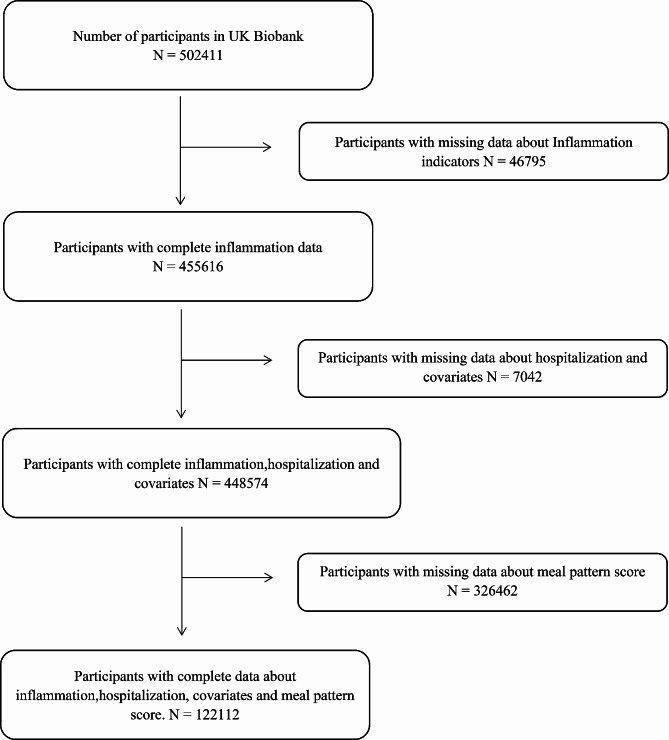
The Flow Diagram in this study

The UK Biobank is a large prospective cohort study that recruited more than 500,000 participants aged 40 to 70 between 2006 and 2010. Details of the UKB study were described previously [21, 22]. After excluding participants without information on inflammatory indicators (*n* = 46,802), age (*n* = 4711), Townsend deprivation index (*n* = 565), and BMI (*n* = 1757) data, a total of 448,574 people were enrolled in this study. The detailed flow diagram is shown in Fig. 1.

Furthermore, to assess the effect of healthy dietary patterns on the association of systemic inflammation with age-related hospitalizations and premature death, we included participants with complete data on both INFLA-Score and dietary information. Among a total of 210 999 participants who completed at least one dietary recall (1–5 times), we excluded 88,887 participants without inflammation data, resulting in a final inclusion of 122,112 participants for subsequent analysis (Fig. 1).

The UK Biobank research received ethical approval from the Northwest Multicenter Research Ethics Committee in the United Kingdom, as well as authorization from the National Information Governance Board for Health and Social Care in England and Wales, and the Community Health Index Advisory Group in Scotland. Written informed consent was obtained from all participants.

### Ascertainment of main exposure

Data on CRP (Field ID: 30,710), Plt (Field ID: 30,080), WBC (Field ID: 30,000), neutrophil count (Field ID: 30,140), and lymphocyte count (Field ID: 30,120) were obtained from baseline blood tests conducted on UKB participants at the first assessment visit. NLR was calculated by neutrophil count/lymphocyte count. Details of data processing can be found on the UKB website (http://biobank.ndph.ox.ac.uk/showcase/showcase/docs/haematology.pdf).

Each inflammatory biomarker level in the population was categorized into quartiles. The INFLA-score was calculated by aggregating equally weighted combinations of the four biomarkers. In this process, each biomarker was further divided into tenths. The highest deciles (6th to 10th) were assigned values ranging from 0 to + 4, while the lowest deciles (1st to 5th) were assigned values from − 4 to 0. Consequently, the INFLA-score ranged from − 16 to 16 and exhibited a normal distribution in the population (Supplementary Fig. 1).

### Ascertainment of main outcomes

Based on a previous study, a total of 23 non-fatal diseases for age-related hospitalizations screening from the 109 types of aging-related diseases, were used in the present study [23].

Death information was accessed through the death certificates held by the National Health Service Information Centre for participants in England and Wales and the National Health Service Central Register Scotland for participants from Scotland. The follow-up person-years were counted from the date of the assessment center visit until the date of loss to follow-up, the date of death, or February 1, 2022, whichever came first. Participants who died at an age younger than 75 were defined as premature death. The International Classification of Diseases-10th Revision (ICD-10) codes were used to identify death type, including cardiovascular disease (I20-25 for CHD and I60-64, I69 for stroke), respiratory disease (J00-J99), cancer (C00-C97), and diabetes (E10-E14).

The Klemera-Doubal method (KDM) biological age (KDM-biological age), an optimal approach for calculating the biology ages, was computed using an R package called “BioAge”, which was trained with 16 indicators including albumin, serum alkaline phosphatase, total cholesterol, creatinine, glycated hemoglobin, systolic blood pressure, diastolic blood pressure, urea, mean corpuscular volume, forced expiratory volume in 1 s, glucose, red blood cell distribution width, gamma-glutamyl transferase, triglycerides, age and BMI (Inflammation-related indicators were excluded). And the biological ages of the whole body and various organs were calculated using an R package called “nhanesR”.

Details of information for age-related hospitalizations, KDM-biological age, whole body, and various organs biological age are provided in Supplementary Material.

### Ascertainment of life expectancy

Firstly, the National Statistical mortality rates for specific sex and age, which were documented in the latest Office for National Statistics life tables from age 40 to age 100 years, were set as the reference mortality value. Secondly, based on the reference mortality value, the sex-specific prevalence of each INFLA-Score, and the HRs of premature death for INFLA-Score (Q2-Q4) group compared to INFLA-Score (Q1) group were used to estimate the survival times at any given age. Finally, the estimated difference in survival time was calculated between the INFLA-Score and reference group. Detailed calculations of life expectancy are provided in Supplementary Material.

### Ascertainment of dietary patterns

The 24-hour dietary recalls were conducted between 2009 and 2012 by the Oxford WebQ to assess 206 types of food and 32 types of beverage intake. The accumulated consumption of each food/beverage item was calculated by multiplying the assigned portion of each item by the amount consumed. The details of dietary pattern scoring including the Dietary Approaches to Stop Hypertension (DASH), the Mediterranean diet (MED), and the Healthy Eating Index-2020 (HEI-2020) are provided in Supplementary Material.

### Covariates assessment

The covariates in this study included age (years), sex (men or women), race (British or other), smoking (previous, current and never), drinking (previous, current and never), Body Mass Index (BMI, ≤ 25 kg/m^2^, 25–30 kg/m^2^ and ≥ 30 kg/m^2^), metabolic equivalent (MET, minutes/week, calculated from the International Physical Activity Questionnaire short form), Townsend deprivation index, health diet group (identified based on a scoring system ranging from 0 to 6 points in total, where a score of three or above was a healthy diet; and the points were assigned as follows: up to 3 times per week for red meat intake, at least four tablespoons of vegetables daily, at least three servings of fruits daily, consumption of fish at least four times per week, at least five servings of grains per week, and a urinary sodium concentration up to 70.6 mmol/L) [24], mineral supplements intake (no or yes), vitamin supplements intake (no or yes), aspirin and other NSAIDs intake (no or yes), medicines for hypercholesterolemia, hypertension and diabetes intake (no or yes), disease status of hypertension, CVD, cancer, diabetes and respiratory diseases (diagnostic records based on ICD-10 codes, I10-I16 for hypertension, I20-25 and I60-64, I69 for CVD, J00-J99 for respiratory disease, C00-C97 for cancer, and E10-E14 for diabetes). This information was collected at the baseline survey.

### Statistical analysis

Baseline characteristics of sociodemographic information, life behaviors, disease status, and drug use were presented as mean ± SD (standard deviation) for continuous variables or numerical (percentage) for categorized variables. And the general linear models and chi-square tests were used to compare the difference by premature mortality status. The inflammatory biomarkers and INFLA-Score were categorized into quartiles. Cox proportional hazards (CPH) models and Restricted cubic spline (RCS) analysis were performed to assess the association of the quartiles of different inflammatory biomarkers and INFLA-Score with aging-related diseases hospitalization and premature death. General linear regression models and line graphs were used to depict the relationships between KDM, whole body, and various organ biological ages of participants across various age groups in different quartiles of INFLA-Score. A series of confounders were adjusted in the CPH, RCS, and general linear regression models, including age, sex, race, smoking, drinking, BMI, MET, Townsend deprivation index, healthy diet group, hypertension, mineral supplements intake, vitamin supplements intake, aspirin and other NSAIDs intake, medicines for hypercholesterolemia, hypertension and diabetes, CVD, Cancer, Diabetes, and Respiratory disease at baseline.

Subgroup analyses were further performed to examine the stability of the results, according to age (≤ 55 or > 55), sex (man or woman), smoking status (never, previous or current), drinking status (no or current), BMI (≤ 25 kg/m^2^, 25–30 kg/m^2^ or ≥ 30 kg/m^2^) and hypertension (no or yes). Three sets of sensitivity analyses were performed to examine whether the results were stable: (1) We excluded participants who had each type of CVD, cancer, diabetes, and respiratory diseases at baseline (*n* = 242,071) and then reanalyzed the results, aiming to eliminate the influence of disease status on the association between the INFLA-Score and age-related hospitalization and premature death in the included population. (2) The INFLA-Score in the entire population was re-divided into quintiles, and then the association between the quintiles of INFLA-Score and age-related health outcomes was assessed, aiming to examine whether the amplitude of the distribution of systemic inflammation status in the population is stable associated with age-related hospitalization and premature death. 3)We excluded participants with a survival time or follow-up duration of less than two years and reanalyzed the results (*n* = 436,624), aiming to correct the impact of severe illness or accidents on these associations.

Moreover, to examine the joint and modification effects of dietary patterns on the association of the INFLA-Score with aging-related diseases hospitalization and premature death, we categorized the score of DASH, MED, and HEI by tertiles. The participants were categorized by cross-grouping the tertile of the dietary pattern score and the quartile of the INFLA-score, and the participants in the lowest tertile of the dietary pattern score and highest quartile of the INFLA-Score were set as the reference group. For the modification effects, the CPH models for the association of the INFLA-score with aging-related diseases hospitalization and premature death were conducted by the tertiles of dietary pattern score.

All statistical analyses were conducted by R 4.2.2, and *p*-values < 0.05 were considered statistically significant.

## Result

### Basic characteristics of participants according to premature death

The differences in baseline characteristics of participants by premature death in terms of sociodemographic information, lifestyle, disease prevalence, and medical conditions, are shown in Table 1. Participants who died prematurely were more likely to be older, female, had higher prevalence of smoking, higher Townsend deprivation index, higher use of aspirin and other NSAIDs, and higher prevalence of hypertension, CVD, cancer, diabetes, and respiratory diseases, and they also had a higher status of BMI and inflammatory biomarkers, but showed lower prevalence of alcohol consumption, MET and healthy diet score.

**Table 1:** Characteristics of participants observed according to whether they died prematurely

Characteristics	Overall	Non-premature death	Premature death	*P*-value
	*N* = 448,574	Q1 *N* = 424,790	Q2 *N* = 23,784	
Age (years)	57 ± 8	56 ± 8	60 ± 6	< 0.001
Sex [N, (%)]				< 0.001
man	242,912 (54%)	233,196 (55%)	9,716 (41%)	
female	205,662 (46%)	191,594 (45%)	14,068 (59%)	
British [N, (%)]	396,909 (88%)	375,460 (88%)	21,449 (90%)	< 0.001
Current smoker [N, (%)]	46,979 (10%)	41,949 (9.9%)	5,030 (21%)	< 0.001
Current drinker [N, (%)]	412,063 (92%)	391,038 (92%)	21,025 (88%)	< 0.001
BMI (kg/m²)	27.4 ± 4.8	27.4 ± 4.7	28.3 ± 5.6	< 0.001
Low MET (minutes/week)	120,982 (27%)	113,761 (27%)	7,221 (30%)	< 0.001
Townsend deprivation index	-1.32 ± 3.08	-1.36 ± 3.06	-0.64 ± 3.43	< 0.001
Health diet group [N, (%)]				< 0.001
no	206,268 (46%)	194,130 (46%)	12,138 (51%)	
yes	242,306 (54%)	230,660 (54%)	11,646 (49%)	
Hypertension [N, (%)]				< 0.001
no	328,905 (73%)	314,643 (74%)	14,262 (60%)	
yes	119,669 (27%)	110,147 (26%)	9,522 (40%)	
Mineral supplements [N, (%)]	192,719 (43%)	183,050 (43%)	9,669 (41%)	< 0.001
Vitamin supplements [N, (%)]	142,131 (32%)	134,738 (32%)	7,393 (31%)	< 0.001
Aspirin and other NSAIDs intake [N, (%)]	198,641 (44%)	186,230 (44%)	12,411 (52%)	< 0.001
Medicines for Hypercholesterolemia,				
Hypertension and diabetes intake [N, (%)]	75,530 (17%)	71,492 (17%)	4,038 (17%)	< 0.001
CVD [N, (%)]				< 0.001
no	279,547 (62%)	268,635 (63%)	10,912 (46%)	
yes	169,027 (38%)	156,155 (37%)	12,872 (54%)	
Cancer [N, (%)]				< 0.001
no	437,662 (98%)	415,563 (98%)	22,099 (93%)	
yes	10,912 (2.4%)	9,227 (2.2%)	1,685 (7.1%)	
Diabetes [N, (%)]				< 0.001
no	419,385 (93%)	399,135 (94%)	20,250 (85%)	
yes	29,189 (6.5%)	25,655 (6.0%)	3,534 (15%)	
Respiratory diseases [N, (%)]				< 0.001
no	407,571 (91%)	386,216 (91%)	21,355 (90%)	
yes	41,003 (9.1%)	38,574 (9.1%)	2,429 (10%)	
C-reactive protein (mg/L)	2.59 ± 4.34	2.51 ± 4.16	4.04 ± 6.55	< 0.001
White blood cell count (cells/Litre)	6.88 ± 2.03	6.84 ± 1.93	7.46 ± 3.34	< 0.001
Platelet count (cells/Litre)	253 ± 60	253 ± 59	251 ± 70	< 0.001
Neutrophill count (cells/Litre)	4.22 ± 1.41	4.20 ± 1.38	4.67 ± 1.75	< 0.001
Lymphocyte count (cells/Litre)	1.96 ± 1.13	1.96 ± 1.00	2.04 ± 2.48	< 0.001

Continuous variables are presented as mean ± SD (standard deviation). Categorical variables are presented as numbers (%, percentage)

BMI, body mass index

### Associations between inflammatory biomarkers, INFLA-score and aging-related hospitalization and premature death

**Fig. 2:**
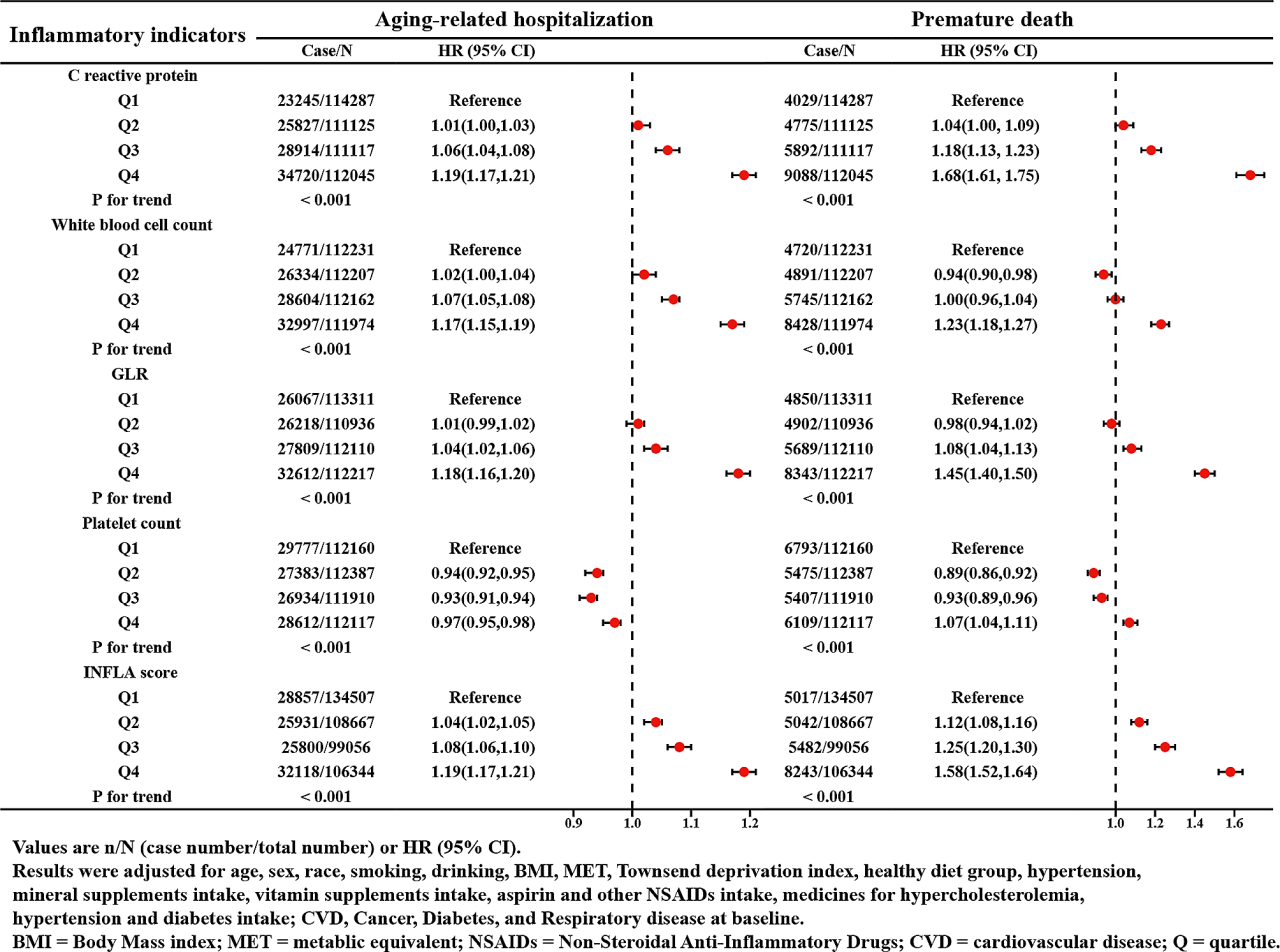
The Association of Inflammatory Indicators and the INFLA-score with Age-related Hospitalization and Premature Death. Values are n/N (case number/total number) or HR (95% CI). Results were adjusted for age, sex, race, smoking, drinking, BMI, MET, Townsend deprivation index, healthy diet group, hypertension, mineral supplements intake, vitamin supplements intake, aspirin and other NSAIDs intake, medicines for hypercholesterolemia, hypertension and diabetes intake; CVD, Cancer, Diabetes, and Respiratory disease at baseline. BMI = Body Mass index; MET = metabolic equivalent; NSAIDs = Non-Steroidal Anti-Inflammatory Drugs; CVD = cardiovascular disease; Q = quartile

After adjustment for multiple covariates, we found a graded relationship between the CRP, WBC, NLR, INFLA-score, and aging-related hospitalization and premature death. As indicated by HR and 95% CI, participants in the highest quartile of the inflammatory biomarkers and the INFLA-score had higher risks of aging-related hospitalizations (HR_CRP_=1.19, 95% CI:1.17–1.21; HR_WBC_=1.17, 95% CI:1.15–1.19; HR_NLR_=1.18, 95% CI:1.16–1.20; HR _INFLA−Score_=1.19, 95% CI:1.17–1.21) and premature death (HR_CRP_=1.68, 95% CI:1.61–1.75; HR _WBC_=1.23, 95% CI: 1.18–1.27; HR_NLR_=1.45, 95% CI: 1.40–1.50; HR _INFLA−Score_ = 1.58, 95% CI: 1.52–1.64) than those in the lowest quartile. Whereas participants in the highest quartile of Plt had lower aging-related hospitalization risk but higher premature death risk (HR _Aging−related hospitalization_ = 0.97, 95% CI: 0.95–0.98; HR _premature death_ = 1.07, 95% CI: 1.04–1.11) than those in the lowest quartile (Fig. 2).

As shown in Supplementary Fig. 1, the RCS curve described a J-shape relationship of the INFLA-Score with the hospitalization of aging-related diseases and premature death (all the *P* for non-linearity < 0.001). Additionally, Table 2 and Supplementary Fig. 2 also showed positive associations of the INFLA-Score with aging-related hospitalization and premature death in different stratified subgroups and sensitivity analyses, further demonstrating the stability of the above results.

**Table 2:** HRs and 95%CI for the association of INFLA-Score with age-related hospitalizations and premature death in stratified population

Subgroups	Aging-related hospitalization					Premature death				
	INFLA-Score					INFLA-Score				
	Quartile 1	Quartile 2	Quartile 3	Quartile 4	*P* for trend	Quartile 1	Quartile 2	Quartile 3	Quartile 4	*P* for trend
Age										
age ≤ 55	ref	1.04(1.01,1.07)	1.08(1.05,1.12)	1.18(1.15,1.22)	< 0.001	ref	1.12(1.03, 1.21)	1.22(1.12, 1.33)	1.53(1.41, 1.66)	< 0.001
age > 55	ref	1.04(1.02,1.06)	1.09(1.07,1.11)	1.20(1.18,1.22)	< 0.001	ref	1.13(1.08,1.18)	1.27(1.22, 1.33)	1.62(1.56,1.69)	< 0.001
Sex										
Man	ref	1.04(1.01,1.06)	1.10(10.7,1.12)	1.23(1.20,1.26)	< 0.001	ref	1.10(1.04,1.16)	1.25(1.19, 1.32)	1.68(1.60,1.77)	< 0.001
Woman	ref	1.04(1.02,1.07)	1.08(1.06,1.11)	1.20(1.17,1.23)	< 0.001	ref	1.11(1.05,1.18)	1.20(1.13, 1.28)	1.47(1.39,1.56)	< 0.001
Smoking status										
never	ref	1.02(1.00,1.05)	1.08(1.06,1.11)	1.19(1.16,1.22)	< 0.001	ref	1.12(1.05, 1.19)	1.16(1.09, 1.23)	1.42(1.34, 1.51)	< 0.001
previous	ref	1.05(1.02,1.07)	1.09(1.06,1.12)	1.22(1.19,1.25)	< 0.001	ref	1.06(1.00,1.13)	1.22(1.15, 1.29)	1.60(1.51,1.70)	< 0.001
current	ref	1.05(1.00,1.10)	1.15(1.09,1.21)	1.26(1.20,1.32)	< 0.001	ref	1.26(1.15,1.37)	1.45(1.33, 1.58)	1.87(1.72,2.04)	< 0.001
Drinking status										
no	ref	1.04(0.97,1.12)	1.13(1.05,1.21)	1.18(1.09,1.27)	< 0.001	ref	1.09(0.91,1.30)	1.23(1.03, 1.47)	1.40(1.17,1.67)	< 0.001
previous	ref	1.06(0.98,1.14)	1.11(1.03,1.20)	1.24(1.15,1.35)	< 0.001	ref	1.21(1.03,1.42)	1.34(1.15, 1.57)	1.60(1.37,1.88)	< 0.001
current	ref	1.04(1.02,1.06)	1.09(1.07,1.11)	1.21(1.19,1.23)	< 0.001	ref	1.11(1.07,1.16)	1.25(1.20, 1.30)	1.59(1.53,1.65)	< 0.001
BMI										
≤ 25 kg/m^2^	ref	1.06(1.02,1.09)	1.10(1.06,1.13)	1.23(1.19,1.27)	< 0.001	ref	1.22(1.13,1.32)	1.30(1.21, 1.41)	1.73(1.61,1.86)	< 0.001
25–30 kg/m^2^	ref	1.02(1.00,1.05)	1.08(1.06,1.11)	1.21(1.18,1.24)	< 0.001	ref	1.09(1.03,1.16)	1.20(1.13, 1.28)	1.56(1.47,1.65)	< 0.001
≥ 30 kg/m^2^	ref	1.04(1.00,1.07)	1.09(1.06,1.12)	1.17(1.14,1.21)	< 0.001	ref	1.11(1.04,1.19)	1.20 (1, 13, 29)	1.44(1.35,1.54)	< 0.001
Hypertension										
no	ref	1.03(1.01,1.05)	1.08(1.06,1.11)	1.21(1.19,1.24)	< 0.001	ref	1.12(1.06, 1.18)	1.26(1.20, 1.32)	1.63(1.55, 1.71)	< 0.001
yes	ref	1.04(1.01,1.07)	1.11(1.08,1.14)	1.23(1.19,1.27)	< 0.001	ref	1.07(1.00,1.14)	1.21(1.14, 1.28)	1.59(1.50,1.69)	< 0.001

Values are n/N (case number/total number) or HR (95% CI). Results were adjusted for age, sex, race, smoking, drinking, BMI, MET, Townsend deprivation index, hypertension, mineral supplements intake, vitamin supplements intake, aspirin and other NSAIDs intake, medicines for hypercholesterolemia, hypertension and diabetes intake; CVD, Cancer, Diabetes, and Respiratory disease at baseline

BMI = Body Mass index; MET = metabolic equivalent; NSAIDs = Non-Steroidal Anti-Inflammatory Drugs; CVD = cardiovascular disease

### Associations between the INFLA-score and cause-specific premature death

**Fig. 3:**
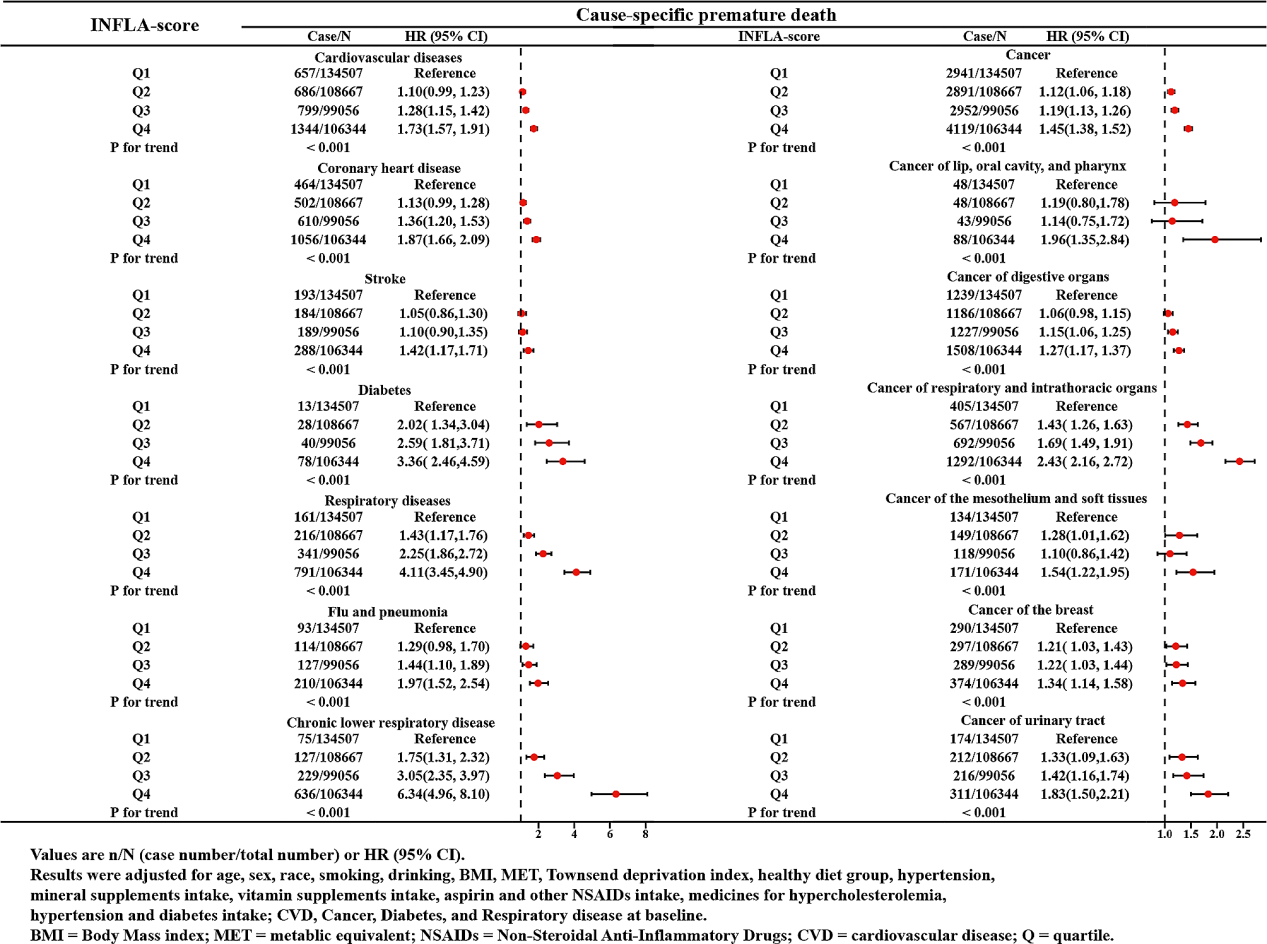
The Association Between the INFLA-score and the Cause-specific Premature death. Values are n/N (case number/total number) or HR (95% CI). Results were adjusted for age, sex, race, smoking, drinking, BMI, MET, Townsend deprivation index, healthy diet group, hypertension, mineral supplements intake, vitamin supplements intake, aspirin and other NSAIDs intake, medicines for hypercholesterolemia, hypertension and diabetes intake; CVD, Cancer, Diabetes, and Respiratory disease at baseline. BMI = Body Mass index; MET = metabolic equivalent; NSAIDs = Non-Steroidal Anti-Inflammatory Drugs; CVD = cardiovascular disease; Q = quartile

Due to CVD, diabetes, respiratory, and cancer diseases accounting for a major percentage of premature death, the relationships between the INFLA-score and cause-specific premature death were further explored. Figure 3 showed that as indicated by HR and 95% CI, participants in the highest quartile of INFLA-score had higher premature death risks of CVD (coronary heart disease and stroke), diabetes, respiratory diseases(flu and pneumonia, and chronic lower respiratory disease), cancer (lip, oral cavity, and pharynx cancers, digestive organ cancers, respiratory and intrathoracic organ cancers, mesothelium and soft tissue cancers, breast cancer, and urinary tract cancer) than those in the lowest quartile (HR_CVD_=1.73, 95% CI: 1.57-1,91; HR _Coronary heart disease_=1.87, 95% CI: 1.66–2.09; HR _Stroke_=1.42, 95% CI: 1.17-1,71; HR _Diabetes_=3.36, 95% CI: 2.46–4.59; HR _Respiratory diseases_=4.11, 95% CI: 3.45–4.90; HR _Flu and pneumonia_=1.97, 95% CI: 1.52–2.54; HR _Chronic lower respiratory disease_=6.34, 95% CI: 4.96–8.10; HR _Cancer_=1.45, 95% CI: 1.38–1.52; HR _Cancer of lip, oral cavity, and pharynx_=1.96, 95% CI: 1.35–2.84; HR _Cancer of the digestive organs_=1.27, 95% CI: 1.17–1.37; HR _Cancer of respiratory and intrathoracic organs_= 2.43, 95% CI: 2.16–2.72; HR _Cancer of the mesothelium and soft tissues_=1.54, 95% CI: 1.22–1.95; HR _Cancer of the breast_=1.34, 95% CI: 1.14–1.58; HR _Cancer of urinary tract_=1.83, 95% CI: 1.50–2.21). And we observed no significant associations between the INFLA-score and other cause-specific premature death, including acute upper respiratory infection, cancer of bone and articular cartilage, melanoma and other skin malignancies, cancer of the female genital organs, cancer of male reproductive organs, cancer of eye, brain, and other central nervous systems, thyroid and other endocrine gland malignancies, cancer of lymphoid, hematopoietic and related tissues (Supplementary Fig. 3).

**Associations between the INFLA-score and biological age of the whole body and specific organs and estimated life expectancy**.

Compared to participants in the lowest quartile of the INFLA-score, participants in the highest quartile of the INFLA-score had a higher biological age of KDM, whole body and specific organs(∆age _KDM_ = 11; ∆age _whole body_ = 5; ∆age _CVD_ = 3; ∆age _kidney_= 3;∆age _liver_ = 5, and all the p-value < 0.001, shown in Supplementary Figs. 4–8 and Supplementary Table 1).

**Fig. 4:**
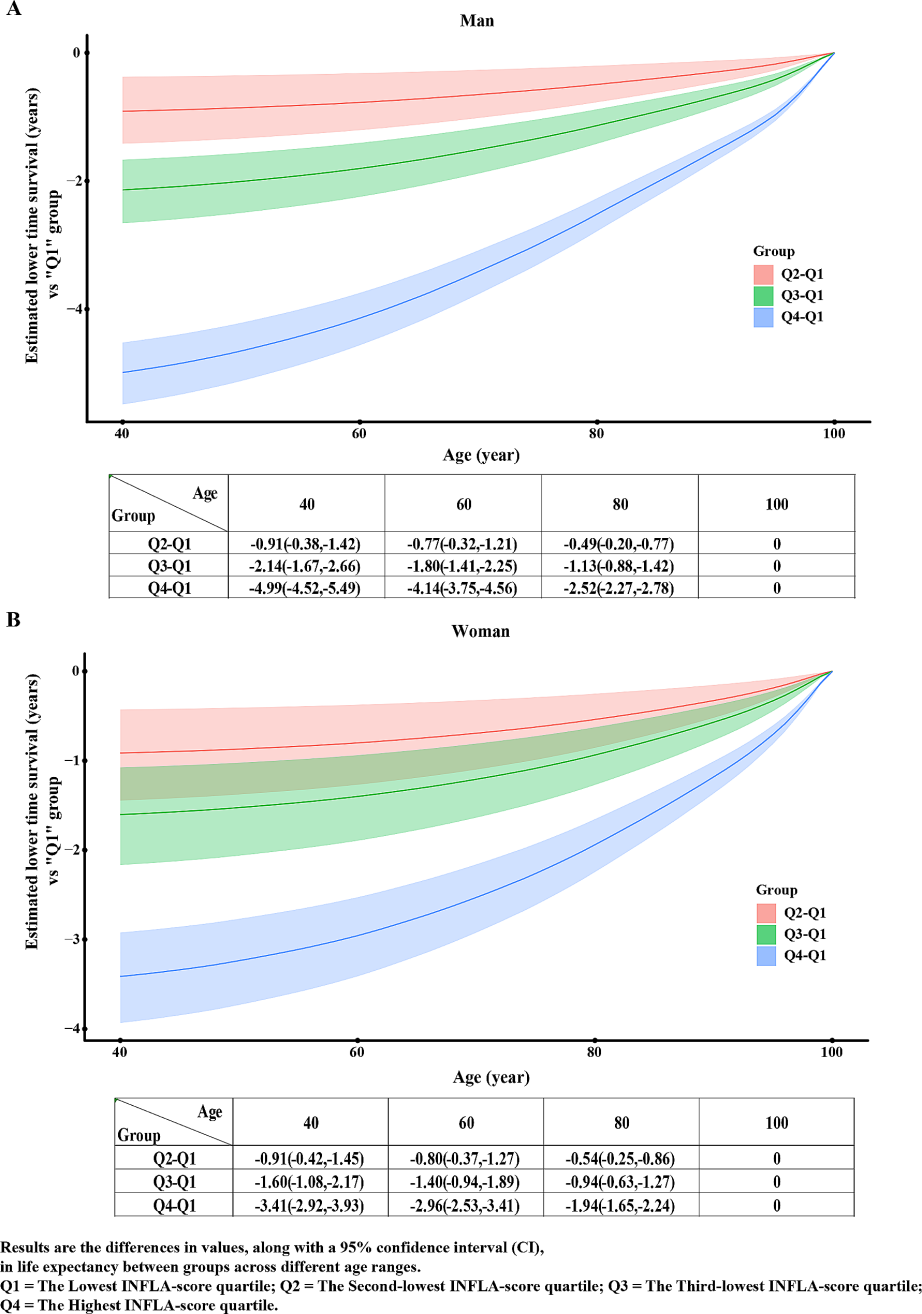
Life Expectancy Disparities Between Other INFLA-Score Quartiles and the Lowest INFLA-Score Quartile for man(*A*) and woman(*B*). Results are the differences in values, along with a 95% confidence interval (CI), in life expectancy between groups across different age ranges. Q1 = The Lowest INFLA-score quartile; Q2 = The Second-lowest INFLA-score quartile; Q3 = The Third-lowest INFLA-score quartile; Q4 = The Highest INFLA-score quartile

And the estimated life expectancy of different quartiles of the INFLA-score is shown in Fig. 4. At age 40, men in the highest quartile of the INFLA-score lost an average of 4.99 (95% CI: 4.52–5.49) years, and women in the highest quartile of the INFLA-score lost an average of 3.41 (95% CI: 2.92–3.93) years of life expectancy than those in the lowest quartile, respectively. The lower life expectancy lost at age 60 was 2.96 (95% CI 2.53–3.41) and 4.14 (95% CI 3.75–4.56) years for women and men, respectively.

### Association of the INFLA-Score with age-related disease hospitalization and premature death in different dietary pattern scores

To examine which dietary pattern had a beneficial impact on preventing aging-related hospitalization and premature death, three types of dietary pattern scores including the HEI-2020, the MED, and the DASH were calculated. We observed inverse associations between each dietary pattern score and risks of aging-related hospitalization and premature death (Supplementary Fig. 11).

**Fig. 5:**
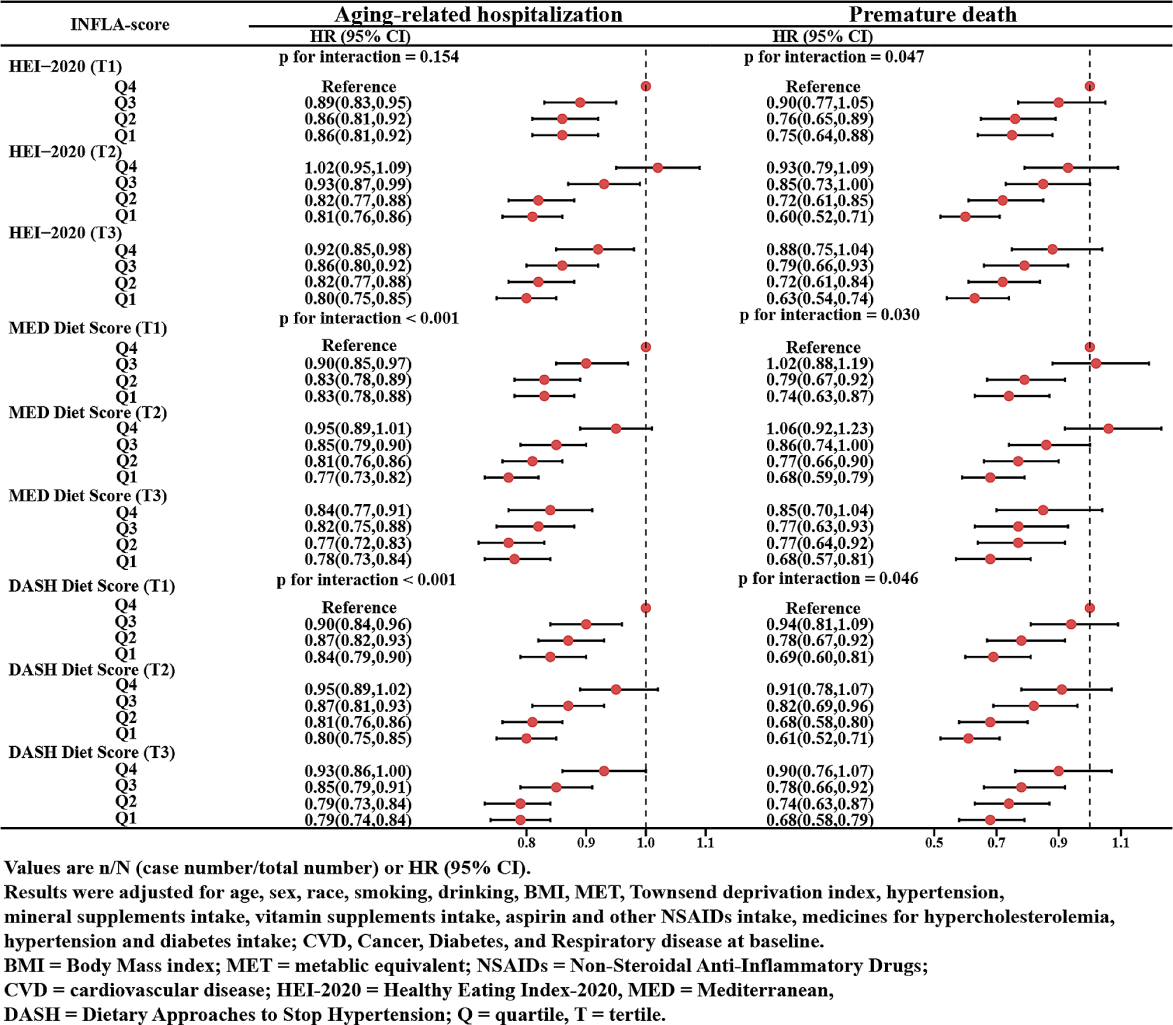
The Joint Association of the INFLA-score and Different Dietary Pattern Scores with Age-related Hospitalization and Premature Death. Values are n/N (case number/total number) or HR (95% CI). Results were adjusted for age, sex, race, smoking, drinking, BMI, MET, Townsend deprivation index, hypertension, mineral supplements intake, vitamin supplements intake, aspirin and other NSAIDs intake, medicines for hypercholesterolemia, hypertension and diabetes intake; CVD, Cancer, Diabetes, and Respiratory disease at baseline. BMI = Body Mass index; MET = metabolic equivalent; NSAIDs = Non-Steroidal Anti-Inflammatory Drugs; CVD = cardiovascular disease. HEI-2020 = Healthy Eating Index-2020, MED = Mediterranean, DASH = Dietary Approaches to Stop Hypertension; Q = quartile; T = tertile

Figure 5 shows the joint effect of the INFLA-Score and these dietary pattern scores on aging-related hospitalization and premature death at different dietary score levels. Compared with the participants in the lowest tertile of dietary pattern score with the highest quartile of INFLA-Score, those in the highest tertile of dietary pattern score with the lowest quartile of INFLA-Score had the lowest risk of aging-related hospitalization (HR_HEI−2020_=0.80, 95% CI: 0.75–0.85; HR_MED_=0.78, 95% CI: 0.73–0.84; HR_DASH_=0.79, 95% CI: 0.74–0.84) and premature death (HR_HEI−2020_=0.63, 95% CI:0.54–0.74; HR_MED_=0.68, 95% CI: 0.57–0.81; HR_DASH_=0.68, 95% CI: 0.58–0.79). Additionally, among the participants with low scores of each dietary pattern, the INFLA-Score was positively associated with aging-related hospitalizations and premature death, however, these associations became non-significant only in participants with high MED dietary pattern score, which indicated a greater effectiveness of MED delaying aging and premature death caused by high inflammation (Table 3).

**Table 3:** HRs and 95%CI for the association of INFLA-score with age-related hospitalizations and premature death in different levels of dietary pattern score

Dietary Pattern	Low					Median					High				
	INFLA-Score					INFLA-Score					INFLA-Score				
	Quartile 1	Quartile 2	Quartile 3	Quartile 4	*P* for trend	Quartile 1	Quartile 2	Quartile 3	Quartile 4	*P* for trend	Quartile 1	Quartile 2	Quartile 3	Quartile 4	*P* for trend
HEI-2020															
Aging-related hospitalization	ref	1.07(1.01,1.15)	1.04(0.97,1.11)	1.18(1.10,1.26)	< 0.001	ref	0.96(0.90,1.02)	1.12(1.05,1.19)	1.22(1.14,1.30)	< 0.001	ref	1.00(0.94,1.07)	1.05(0.98,1.12)	1.15(1.07,1.23)	< 0.001
Premature death	ref	1.09(0.92,1.29)	1.18(1.00,1.38)	1.38(1.16,1.63)	< 0.001	ref	1.16(0.97,1.38)	1.39(1.17,1.65)	1.57(1.32,1.85)	< 0.001	ref	0.97(0.81,1.16)	1.21(1.02,1.43)	1.30(1.09,1.56)	< 0.001
MED															
Aging-related hospitalization	ref	1.00(0.93,1.06)	1.07(1.00,1.14)	1.19(1.11,1.27)	< 0.001	ref	1.03(0.97,1.09)	1.10(1.04,1.17)	1.21(1.14,1.28)	< 0.001	ref	0.99(0.91,1.07)	0.99(0.91,1.07)	1.07(0.99,1.16)	0.108
Premature death	ref	1.10(0.93,1.30)	1.35(1.15,1.58)	1.38(1.16,1.63)	< 0.001	ref	1.04(0.89,1.21)	1.31(1.13,1.52)	1.47(1.27,1.70)	< 0.001	ref	0.96(0.77,1.20)	1.18(0.95,1.46)	1.19(0.96,1.47)	0.058
DASH															
Aging-related hospitalization	ref	1.07(1.00,1.13)	1.09(1.02,1.16)	1.20(1.12,1.28)	< 0.001	ref	0.99(0.93,1.06)	1.07(1.00,1.14)	1.20(1.12,1.28)	< 0.001	ref	0.95(0.89,1.02)	1.03(0.96,1.10)	1.14(1.07,1.22)	< 0.001
Premature death	ref	1.19(1.01,1.41)	1.41(1.19,1.67)	1.50(1.27,1.78)	< 0.001	ref	1.09(0.91,1.30)	1.37(1.15,1.64)	1.55(1.30,1.85)	< 0.001	ref	0.94(0.79,1.13)	1.14(0.96,1.35)	1.23(1.03,1.45)	0.005

Values are n/N (case number/total number) or HR (95% CI). Results were adjusted for age, sex, race, smoking, drinking, BMI, MET, Townsend deprivation index, hypertension, mineral supplements intake, vitamin supplements intake, aspirin and other NSAIDs intake, medicines for hypercholesterolemia, hypertension and diabetes intake; CVD, Cancer, Diabetes, and Respiratory disease at baseline

BMI = Body Mass index; MET = metabolic equivalent; NSAIDs = Non-Steroidal Anti-Inflammatory Drugs; CVD = cardiovascular disease

HEI-2020 = Healthy Eating Index, MED = Mediterranean, DASH = Dietary Approaches to Stop Hypertension

## Discussion

In the UK biobank prospective cohort, this study observed a significant association between a higher systemic inflammation state and various aging parameters, including an elevated biologic age, increased risks of age-related hospitalization, and total as well as cause-specific premature death. Notably, the higher systemic inflammation level was found to potentially reduce lifespan by 4.99 years for men and 3.41 years for women at age 40, as well as by 4.14 years for men and 2.96 years for women at age 60. Additionally, the Mediterranean (MED) dietary pattern was identified as an effective measure for alleviating the risks of age-related hospitalization and premature death associated with elevated systemic inflammation.

Current evidence indicates that the systemic inflammation level often accompanies the aging process in organisms, tightly correlating with various pathological disorders such as the loss of lean body mass, reduction of immune function, and increased risk of chronic disease [6, 25]. Previous experimental studies have indicated that inflammatory markers, including CRP, WBC, NLR and Plt, serve as major risk factors in promoting cell senescence and DNA damage, decreasing telomerase activity and mitochondrial metabolic function [26–29]. Therefore, numerous epidemiological studies have focused on examining the relationship between a single inflammatory marker and cause-specific or all-cause mortality [30, 31]. However, these associations were found to be unstable, potentially because a single inflammatory marker probably may not adequately reflect the integrated systemic inflammatory status in relation to aging-related health outcomes. Supporting this hypothesis, our study also observed that participants with high Plt levels had a higher risk of premature death but a reduced risk of age-related hospitalization.

It has been documented that systemic inflammatory response plays a crucial role in mediating sub-clinical and clinical events [32–34]. Therefore, the INFLA-score, serving as a comprehensive index, can be considered as a unique method for evaluating the association between the integrated systemic inflammatory response and age-related health outcomes [35]. Bonaccio et al. reported that an increased INFLA-score was associated with a 44% increased risk of all-cause mortality in a large cohort study [36]. Consistent with previous studies, we found that a higher INFLA-score served as a strong and robust contributor to an increased risk of age-related hospitalization and premature death. Furthermore, we also observed a positive association between INFLA-score and the risks of premature death from CVD, diabetes, respiratory diseases, and cancer. Our data underscored the substantial contribution of a complex inflammatory response to disease development, emphasizing the importance of research in the field of systemic inflammation and human health. In addition, the present study showed that participants in the highest quartile of the INFLA-score were associated with a significantly lower life expectancy for women and men at any ages compared to those in the lowest quartile. Further, we revealed that increased biological age, disturbance in homeostasis, and declined function were potential mechanisms contributing to the acceleration of the aging process and premature death. Hence, effectively reducing inflammatory status emerges as a potential strategy for promoting health and longevity.

Increasing evidence has proven that adhering to a healthy dietary pattern can effectively reduce circulating inflammation levels [37–41]. Consistent with previous studies, our study also observed that the classical dietary patterns including DASH, MED and HEI-2020 were all inversely associated with risk of age-related hospitalization and premature death. Compared with previous studies, we found that the association between higher systemic inflammation levels and increased risks of aging-related diseases, hospitalization, and premature death significantly attenuated only among the participants with higher adherence to the MED dietary pattern, suggesting that the MED dietary pattern may serve as an effective dietary intervention strategy for delaying aging process and extending lifespan. A series of previous studies may provide potential biological mechanisms underlying the above observations. Mechanistically, the distinctive feature of the Mediterranean (MED) diet involves an elevated consumption of unsaturated fatty acids. These fatty acids have the capacity to inhibit the toll receptor signaling pathway in immune cells, resulting in a reduction in the transcription of inflammatory factors and systematic inflammation levels [42, 43]. In particular, ω-3 fatty acids, such as EPA and DHA, can contribute to the inhibition of prostaglandin and leukotriene production, thereby alleviating inflammatory responses [44, 45]. Additionally, unsaturated fatty acids could also generate specific metabolites, including Resolvin E3 and Resolvin D5, which possess anti-inflammatory effects and contribute to the inhibition of macrophage activity [46, 47]. Based on the findings of this study, future investigations are warranted to explore the beneficial effect of healthy dietary pattern on inflammatory response for providing more accurate dietary guidance for various aging-related diseases.

This study has several public health implications. Firstly, this population-based study used a novel assessment method of the INFLA-score to examine the association between the systemic inflammation level and age-related health outcomes, and consistent results were also observed in several sensitivity and subgroup analyses. Secondly, our findings indicate that dietary intervention is an effective measure to reduce systemic inflammation, delaying aging processing and preventing premature death. However, we also recognized that our study had certain limitations. Firstly, we could not completely exclude the possibility that participants with high INFLA-score were related to various diseases. Although the sensitive analysis indicated that the positive associations between the INFLA-score and risk of age-related hospitalization and premature mortality were robust among participants without CVD, cancer, diabetes and respiratory diseases at baseline. Secondly, although the UK Biobank study investigated the dietary components consumption with a multi-repeated method, participants might change their eating habits over time. Therefore, future study is needed to evaluate the longitudinal effect of healthy dietary pattern on the association between systemic inflammation and the aging process. Thirdly, this study is observational in nature. Although we controlled a series of confounders that were related to the aging process and premature death, we cannot rule out the unmeasured variables that may influence our findings. Fourthly, this study was conducted only based on the population in the UK, limiting the generalization of these findings to other populations. Future studies conducted on the populations from other regions are still needed.

## Conclusion

The integrated systemic inflammatory state was positively associated with biological age, frailty index, and elevated risk of age-related hospitalization and premature death, while adhering to a MED dietary pattern could more effectively ameliorate the adverse effects of higher systemic inflammation.

### Electronic supplementary material

Below is the link to the electronic supplementary material.

Supplementary Material 1

Supplementary Material 2

Supplementary Material 3

Supplementary Material 4

Supplementary Material 5

Supplementary Material 6

Supplementary Material 7

Supplementary Material 8

Supplementary Material 9

Supplementary Material 10

Supplementary Material 11

## Data Availability

The data supporting the findings of this study from the UK Biobank cohort are accessible through the UK Biobank project site, following a successful registration and application process. More information can be found at https://www.ukbiobank.ac.uk/.
