# Supplementary figures and images for: The association of circulating systemic inflammation with premature death and the protective role of the Mediterranean diet: a large prospective cohort study of UK biobank
Source: BMC Public Health. 2024 May 30;24:1449. doi: 10.1186/s12889-024-18888-x (PMC11312373; doi:10.1186/s12889-024-18888-x)

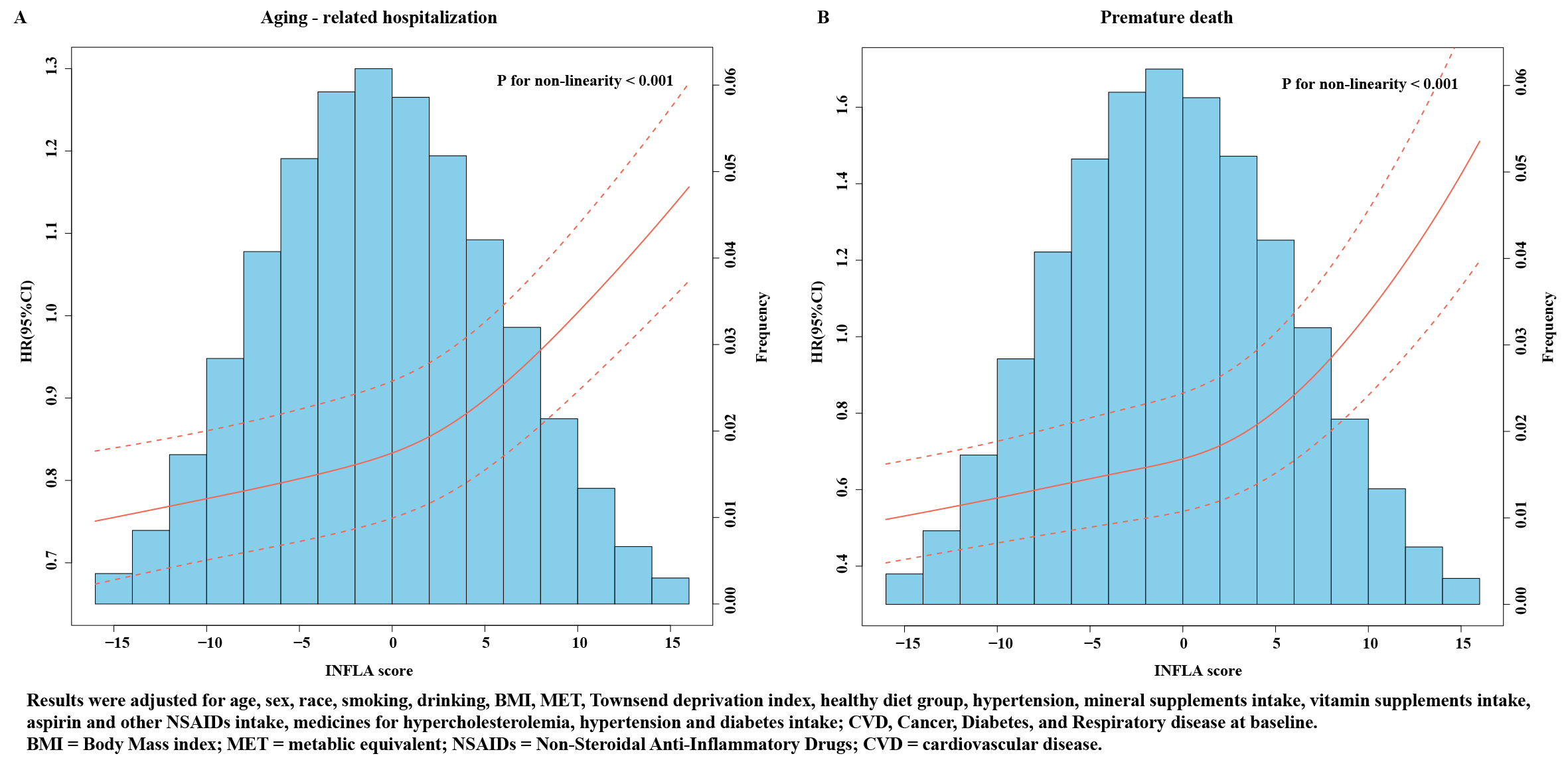

Supplement: Supplementary file 1 — Supplementary Material 1 [file 12889_2024_18888_MOESM1_ESM.tif]

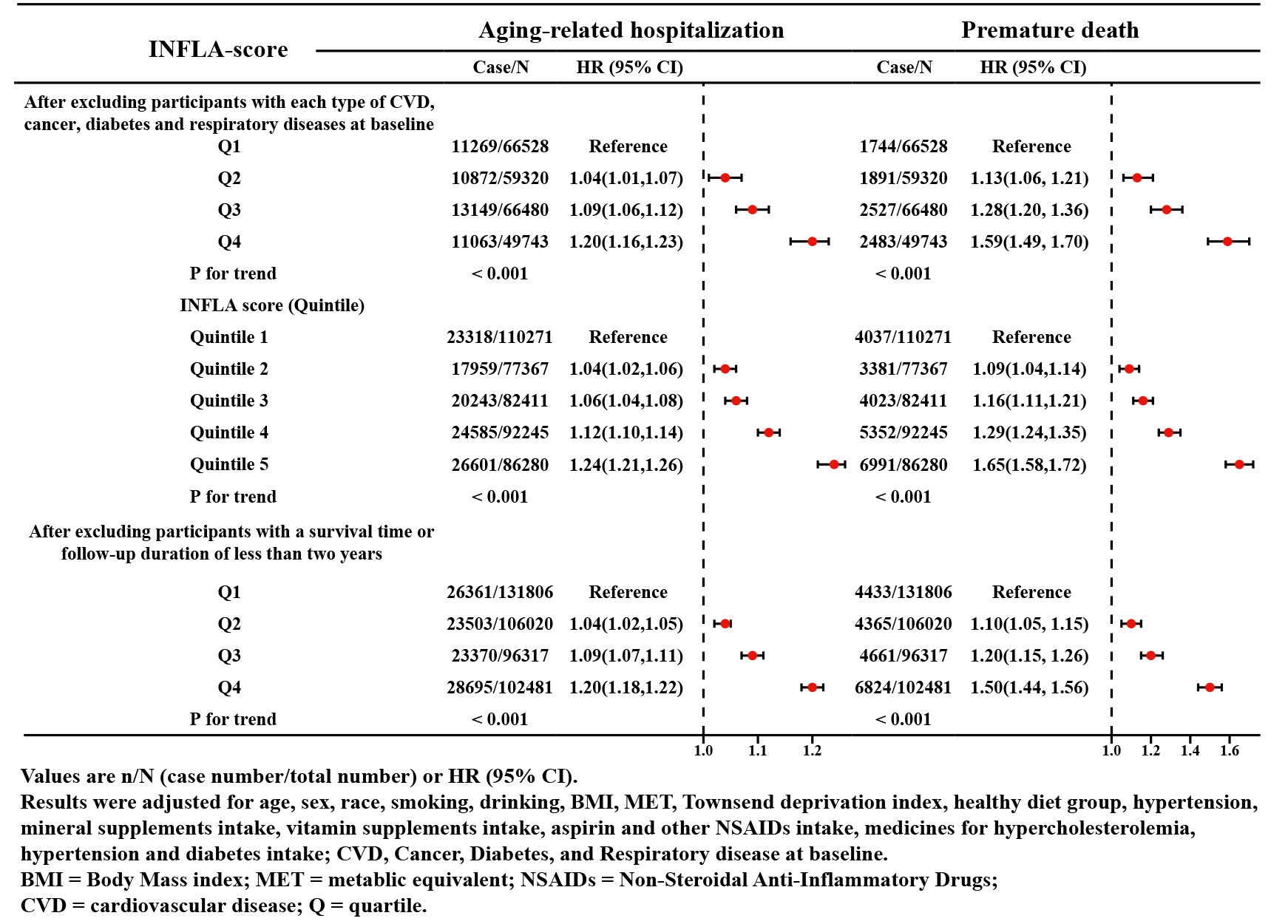

Supplement: Supplementary file 2 — Supplementary Material 2 [file 12889_2024_18888_MOESM2_ESM.tif]

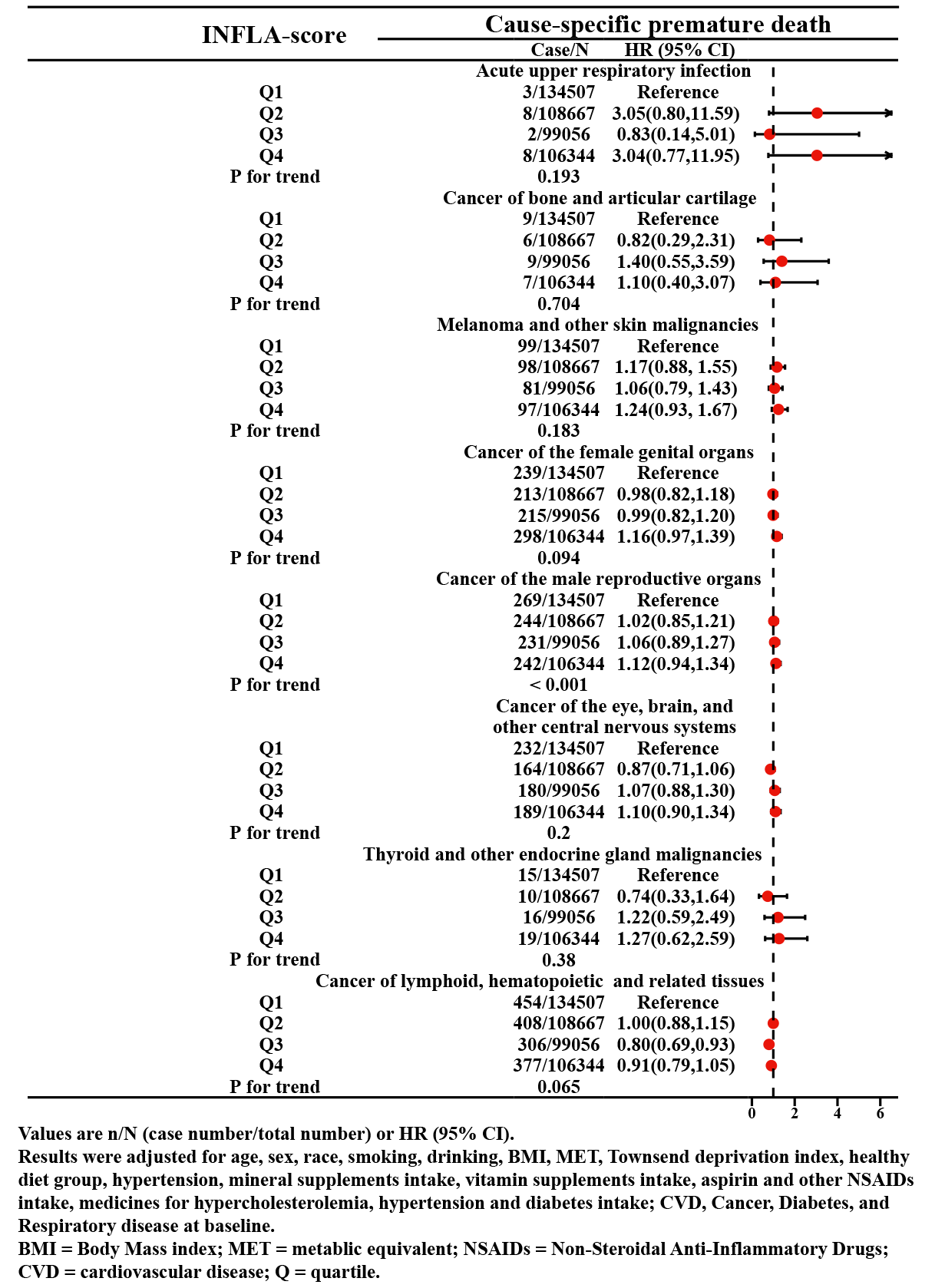

Supplement: Supplementary file 3 — Supplementary Material 3 [file 12889_2024_18888_MOESM3_ESM.tif]

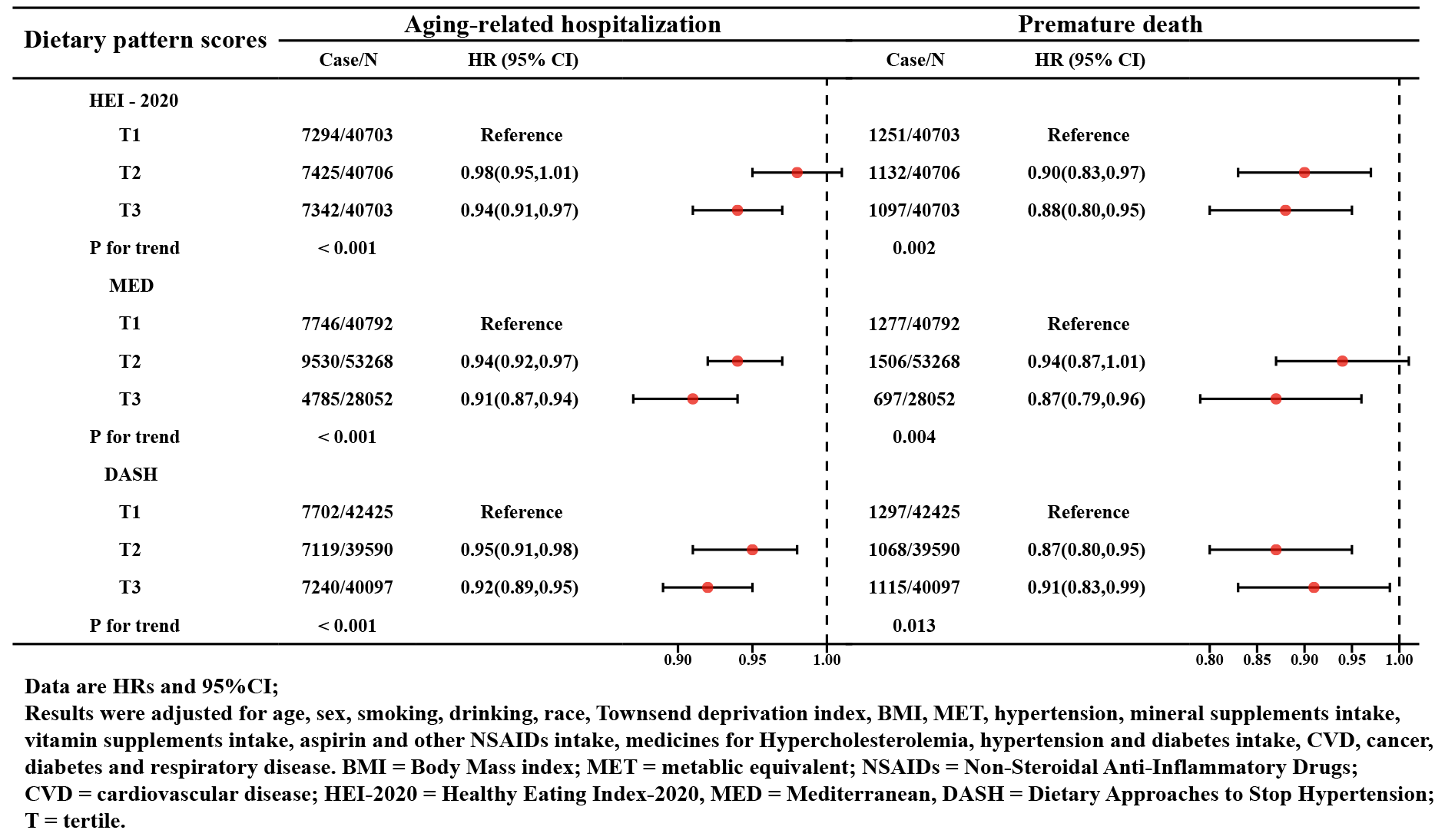

Supplement: Supplementary file 9 — Supplementary Material 9 [file 12889_2024_18888_MOESM9_ESM.tif]
