# Supplementary material for: The association of circulating systemic inflammation with premature death and the protective role of the Mediterranean diet: a large prospective cohort study of UK biobank
Source: BMC Public Health. 2024 May 30;24:1449. doi: 10.1186/s12889-024-18888-x (PMC11312373; doi:10.1186/s12889-024-18888-x)
Supplement: Supplementary file 11 — Supplementary Material 11 [file 12889_2024_18888_MOESM11_ESM.docx]

**Supplementary materials**

**1 Aging-related hospitalizations**

**1.1 Details of the ICD-10 codes for the final 23 age-related diseases**

|  | ICD code | ICD Description |
| --- | --- | --- |
| 1 | R31 | Unspecified hematuria |
| 2 | E86 | Volume depletion |
| 3 | R54 | Senility |
| 4 | F03 | Unspecified dementia |
| 5 | G20 | Parkinson's disease |
| 6 | R55 | Syncope and collapse |
| 7 | L97 | Ulcer of lower limb, not elsewhere classified |
| 8 | N19 | Unspecified renal failure |
| 9 | R33 | Retention of urine |
| 10 | R69 | Unknown and unspecified causes of morbidity |
| 11 | R32 | Unspecified urinary incontinence |
| 12 | Y95 | Nosocomial condition |
| 13 | R02 | Gangrene, not elsewhere classified |
| 14 | R13 | Dysphagia |
| 15 | F10 | Mental and behavioral disorders due to use of alcohol |
| 16 | F32 | Depressive episode |
| 17 | R11 | Nausea and vomiting |
| 18 | W18 | Other fall on same level |
| 19 | L89 | Decubitus ulcer |
| 20 | G40 | Epilepsy |
| 21 | E05 | Thyrotoxicosis |
| 22 | A09 | Diarrhea and suspected infectious gastroenteritis |
| 23 | J22 | Unexplained acute lower respiratory tract infection |

**Supplemental reference**

1. Chan MS, Arnold M, Offer A, et al. A Biomarker-based Biological Age in UK Biobank: Composition and Prediction of Mortality and Hospital Admissions. *J Gerontol A Biol Sci Med Sci*. 2021;76(7):1295-1302. doi:10.1093/gerona/glab069

**2 Biomarkers of the whole body and various organs biological age**

A multiple linear regression model was employed to calculate the biological ages, with the actual age as the dependent variable and numerous biomarkers as independent variables. The intercept and beta coefficient of each biomarker were further utilized to measure biological ages.

**2.1** **Whole body - Biological age**

In the UK Biobank data study, the calculation of the whole body-biological age involves initially screening from 72 commonly used physical or biochemical markers. Subsequently, markers with missing values exceeding 10% or those without a linear trend with the actual age were excluded. After excluding inflammation-related indicators, a total of 42 biomarkers were ultimately used, including systolic blood pressure, diastolic blood pressure, pulse, apolipoprotein A, apolipoprotein B, High Density Lipoprotein (HDL), Low Density Lipoprotein (LDL), triglycerides, glucose, HemoglobinA1c (HbAlc), insulin like growth factor (IGF), albumin, alanine amino transferase, aspartate amino transferase, direct bilirubin, total bilirubin, Gamma glutamyl transferase, standing height, waist to hip ratio, Whole body fat free mass, basal metabolic rate, left hand grip, right hand grip, alkaline phosphatase, vitamin D, time to complete round, mean time to correctly identify matches, number of incorrect matches in round, hemoglobin concentration, Immature reticulocyte fraction, mean corpuscular volume, mean reticulocyte volume, mean sphered cell volume, sodium urine, creatinine enzymatic urine, urea, creatinine, cystatin C, total protein, urate, Forced expiratory volume in 1second (FEV1) and Forced vital capacity (FVC).

**2.2 Various organs - Biological age ^[1]^**

**Cardiovascular system**

Triglyceride, low density lipoprotein, fasting blood glucose, high density lipoprotein, systolic pressure, diastolic pressure and total cholesterol.

**Kidney**

Serum creatinine and serum uric acid.

**Liver**

Glutamyl transpeptidase, serum aspartate aminotransferase, serum alanine aminotransferase and albumin concentration.

**Supplemental reference**

1. Xing W, Gao W, Zhao Z, et al. Dietary flavonoids intake contributes to delay biological aging process: analysis from NHANES dataset. J Transl Med. 2023;21(1):492. Published 2023 Jul 21. doi:10.1186/s12967-023-04321-1

**3 Life expectancy ^[1-4]^**

**3.1 Disease Details Required for Frailty Index Calculation**

The National Statistical mortality rates for specific sex and age, which were documented in the latest Office for National Statistics life tables from age 40 to age 100 years, was set as the reference mortality value.

After that, based on the reference mortality value, the sex-specific prevalence of each INFLA-Score, and the HRs of premature death for INFLA-Score (Q2-Q4) group compared to INFLA-Score (Q1) group were used to estimate the survival times at any given age, based on the following sets:

1. Obtaining HRs stratified by sex for INFLA-Score and premature death.
2. Obtaining the frequency for each quartile of the INFLA-Score.
3. Calculate the mortality rate of the INFLA-Score (Q1) group according to the equation: IRreference = IRa/(𝑝reference+ ∑3𝑝𝑗 × H𝑅𝑗)

IRreference: mortality in the INFLA-Score (Q1) group, IRa: Sex- and age-specific mortality rates provided by life tables, 𝑝𝑗: the frequency of each group, H𝑅𝑗: HR value results for INFLA-Score (Q2-Q4) group.

4) Mortality rate in the other groups equals the death rate in the IRreference group multiplied by the HR value for each group. And IRao=mortality rate.

Finally, the estimated difference in survival time was calculated between INFLA-Score groups, based on the following sets:

1. Assuming a population of 100,000 for the 40-year-old group, the calculation includes determining the number of deaths (dx) and the number of survivors (lx) for each age group. The formulas are dx＝lx×IRao；lx+1=lx－dx.
2. The calculation involves determining the person-years survived (Lx) for each age group and the total person-years survived (Tx). Person-years survived represent the cumulative survival years for individuals aged X and above in the coming year. The formulas are Lx=1/2(lx+lx+1)；Tx=∑Lx.
3. The average life expectancy (ex) ＝Tx/lx.

Life expectancy details are provided in Supplementary Table 2.

**Supplemental reference**

1. Arias E. United States life tables, 2008. Natl Vital Stat Rep. 2012 Sep 24;61(3):1-63

2. Chiang CL, World Health Organization. Life table and mortality analysis. 1979. Publisher: Geneva : World Health Organization

3. Woloshin S, Schwartz LM, Welch HG. The risk of death by age, sex, and smoking status in the United States: putting health risks in context. J Natl Cancer Inst 2008;100(12):845-53

4. Ma H, Xue Q, Wang X, et al. Adding salt to foods and hazard of premature mortality. Eur Heart J. 2022;43(30):2878-2888. doi:10.1093/eurheartj/ehac208

**4 Meal Pattern Score**

4.1 Healthy Eating Index ^[1,2]^

The HEI-2020 consists of 13 sections with a maximum total score of 100 points. The total score is the sum of the scores for the adequacy component (ie, foods that are eaten in excess for good health) and the moderate component (ie, foods that are limited for good health). Each ingredient is scored on a 1,000-calorie density basis, with the exception of fatty acids, which are the ratio of unsaturated to saturated fatty acids. See the attached table for details of the HEI-2020 scoring criteria.

HEI–2020 Components & Scoring Standards

| Component | Maximum points | Standard for maximum score | Standard for minimum score of zero |
| --- | --- | --- | --- |
| Adequacy: |  |  |  |
| [Total Fruits](https://epi.grants.cancer.gov/hei/developing.html#f2b)^2^ | 5 | ≥0.8 cup equiv. per 1,000 kcal | No Fruits |
| [Whole Fruits](https://epi.grants.cancer.gov/hei/developing.html#f3b)^3^ | 5 | ≥0.4 cup equiv. per 1,000 kcal | No Whole Fruits |
| [Total Vegetables](https://epi.grants.cancer.gov/hei/developing.html#f4b)^4^ | 5 | ≥1.1 cup equiv. per 1,000 kcal | No Vegetables |
| [Greens and Beans](https://epi.grants.cancer.gov/hei/developing.html#f4b)^4^ | 5 | ≥0.2 cup equiv. per 1,000 kcal | No Dark Green Vegetables or Legumes |
| Whole Grains | 10 | ≥1.5 oz equiv. per 1,000 kcal | No Whole Grains |
| [Dairy](https://epi.grants.cancer.gov/hei/developing.html#f5b)^5^ | 10 | ≥1.3 cup equiv. per 1,000 kcal | No Dairy |
| [Total Protein Foods](https://epi.grants.cancer.gov/hei/developing.html#f6b)^4^ | 5 | ≥2.5 oz equiv. per 1,000 kcal | No Protein Foods |
| Seafood and Plant Proteins^5^ | 5 | ≥0.8 oz equiv. per 1,000 kcal | No Seafood or Plant Proteins |
| [Fatty Acids](https://epi.grants.cancer.gov/hei/developing.html#f7b)^7^ | 10 | (PUFAs + MUFAs)/SFAs ≥2.5 | (PUFAs + MUFAs)/SFAs ≤1.2 |
| Moderation: | | | |
| Refined Grains | 10 | ≤1.8 oz equiv. per 1,000 kcal | ≥4.3 oz equiv. per 1,000 kcal |
| Sodium | 10 | ≤1.1 gram per 1,000 kcal | ≥2.0 grams per 1,000 kcal |
| Added Sugars | 10 | ≤6.5% of energy | ≥26% of energy |
| Saturated Fats | 10 | ≤8% of energy | ≥16% of energy |
| Score | 0-100 points total (13 components: 0–5 points or 0-10 points each) | | |

1: The total HEI score is the sum of the adequacy components (i.e. foods to eat more of for good health) and moderation components (i.e. foods to limit for good health).

2: Includes 100% fruit juice.

3: Includes all forms except juice.

4: Includes legumes (beans and peas).

5: Includes all milk products, such as fluid milk, yogurt, and cheese, and fortified soy beverages.

6: Includes seafood, nuts, seeds, soy products (other than beverages), and beans, peas, and lentils.

7: Ratio of poly- and monounsaturated fatty acids (PUFAs and MUFAs) to saturated fatty acids (SFAs).

4.2 Mediterranean diet ^[3]^

The Mediterranean diet score included nine components and awarded 1 point for an intake equal to or above the cohort specific median for vegetables, fruits, whole grains, nuts, legumes, fish, and ratio of monounsaturated to saturated fat and 1 point for an intake below the cohort specific median for red and processed meat and for alcohol intake 5-15 g/d for women and 10-25 g/d for men. The total score ranged from 0 to 9 points. Mediterranean dietary patterns are divided according to the degree of different food groups in the table below.

Mediterranean diet score Components & Scoring Standards

| Component | 1 point for individuals who meet each standard |
| --- | --- |
| vegetables | Above or equal to the median |
| fruits | Above or equal to the median |
| whole grains | Above or equal to the median |
| nuts | Above or equal to the median |
| legumes | Above or equal to the median |
| fish | Above or equal to the median |
| ratio of monounsaturated to saturated fat | Above or equal to the median |
| red and processed meat | below median |
| alcohol intake | 5-15 g/d for women and 10-25 g/d for men. |
| Score | 0-9 points total |

4.3 Dietary Approaches to Stop Hypertension (DASH) ^[4]^

The DASH dietary pattern focuses on eight key foods or nutrient components: high intakes of fruits, vegetables, nuts and legumes, low-fat dairy and whole grains, and low intakes of sodium, sweetened beverages, and red and processed meats kind.

DASH diet score Components & Scoring Standards

| Components | 5 points for individuals who meet each standard |
| --- | --- |
| Vegetables | Excluding potatoes: highest quintile |
| Fruit | Total fruit (all fruits and fruit juices): highest quintile |
| Nuts and legumes | Nuts, seeds, and legumes: highest quintile |
| Whole grains | Highest quintile |
| Dairy | Low-fat dairy: highest quintile |
| Red and processed meat | Lowest quintile |
| SSBs and fruit juice | Lowest quintile |
| Sodium | Lowest quintile |
| Score | 8–40 points total (8 components: 1–5 points each) |

**Supplemental reference**

1. https://epi.grants.cancer.gov/hei/developing.html#2020

2. Shams-White MM, Pannucci TE, Lerman JL, et al. Healthy Eating Index-2020: Review and Update Process to Reflect the Dietary Guidelines for Americans,2020-2025. J Acad Nutr Diet. 2023;123(9):1280-1288.

3. Trichopoulou A, Costacou T, Bamia C, Trichopoulos D. Adherence to a Mediterranean diet and survival in a Greek population. N Engl J Med. 2003;348(26):2599-2608.

4. Fung TT, Chiuve SE, McCullough ML, Rexrode KM, Logroscino G, Hu FB. Adherence to a DASH-style diet and risk of coronary heart disease and stroke in women [published correction appears in Arch Intern Med. 2008 Jun 23;168(12):1276].

**Supplementary Table 1 Observed biological age characteristics of participants according to different INFLA score quartiles.**

| Variables | INFLA score | | | | |
| --- | --- | --- | --- | --- | --- |
|  | Q1 | Q2 | Q3 | Q4 | p-value |
| KDM biological age | 58±14 | 62±14 | 65±15 | 69±16 | <0.001 |
| Whole body biological age | 54±10 | 56±10 | 57±10 | 59±11 | <0.001 |
| Cardiovascular system biological age | 55±11 | 56±11 | 57±11 | 58±11 | <0.001 |
| Kidney biological age | 55±10 | 56±11 | 57±12 | 58±13 | <0.001 |
| Liver biological age | 54±21 | 56±21 | 57±23 | 59±25 | <0.001 |
| Continuous variables are presented as mean ± SD (standard deviation). | | | | | |

**Supplementary Figure legend**

Supplementary Figure 1. The INFLA-Score Distribution and its Association with Age-Related Hospitalization and Premature Death in the Population.

Results were adjusted for age, sex, race, smoking, drinking, BMI, MET, Townsend deprivation index, healthy diet group, hypertension, mineral supplements intake, vitamin supplements intake, aspirin and other NSAIDs intake, medicines for hypercholesterolemia, hypertension and diabetes intake; CVD, Cancer, Diabetes, and Respiratory disease at baseline.

BMI = Body Mass index; MET = metabolic equivalent; NSAIDs = Non-Steroidal Anti-Inflammatory Drugs; CVD = cardiovascular disease.

Supplementary Figure 2. Sensitivity Analysis of the Association Between the INFLA-Score and Age-Related Hospitalizations and Premature Death.

Values are n/N (case number/total number) or HR (95% CI). Results were adjusted for age, sex, race, smoking, drinking, BMI, MET, Townsend deprivation index, healthy diet group, hypertension, mineral supplements intake, vitamin supplements intake, aspirin and other NSAIDs intake, medicines for hypercholesterolemia, hypertension and diabetes intake; CVD, Cancer, Diabetes, and Respiratory disease at baseline.

BMI = Body Mass index; MET = metabolic equivalent; NSAIDs = Non-Steroidal Anti-Inflammatory Drugs; CVD = cardiovascular disease; Q=quartile.

Supplementary Figure 3. The Association Between the INFLA-score and Other Cause-specific premature death.

Values are n/N (case number/total number) or HR (95% CI). Results were adjusted for age, sex, race, smoking, drinking, BMI, MET, Townsend deprivation index, healthy diet group, hypertension, mineral supplements intake, vitamin supplements intake, aspirin and other NSAIDs intake, medicines for hypercholesterolemia, hypertension and diabetes intake; CVD, Cancer, Diabetes, and Respiratory disease at baseline.

BMI = Body Mass index; MET = metabolic equivalent; NSAIDs = Non-Steroidal Anti-Inflammatory Drugs; CVD = cardiovascular disease; Q = quartile.

Supplementary Figure 4. The KDM Biological Age of Participants Across Various Age Groups in Different Quartiles of the INFLA-Score.

Q1 = The Lowest INFLA-score quartile; Q2 = The Second-lowest INFLA-score quartile; Q3 = The Third-lowest INFLA-score quartile; Q4 = The Highest INFLA-score quartile.

Supplementary Figure 5. The Whole Body Biological Age of Participants Across Various Age Groups in Different Quartiles of the INFLA-Score.

Q1 = The Lowest INFLA-score quartile; Q2 = The Second-lowest INFLA-score quartile; Q3 = The Third-lowest INFLA-score quartile; Q4 = The Highest INFLA-score quartile.

Supplementary Figure 6. The Cardiovascular System Biological Age of Participants Across Various Age Groups in Different Quartiles of the INFLA-Score.

Q1 = The Lowest INFLA-score quartile; Q2 = The Second-lowest INFLA-score quartile; Q3 = The Third-lowest INFLA-score quartile; Q4 = The Highest INFLA-score quartile.

Supplementary Figure 7. The Kidney Biological Age of Participants Across Various Age Groups in Different Quartiles of the INFLA-Score.

Q1 = The Lowest INFLA-score quartile; Q2 = The Second-lowest INFLA-score quartile; Q3 = The Third-lowest INFLA-score quartile; Q4 = The Highest INFLA-score quartile.

Supplementary Figure 8. The Liver Biological Age of Participants Across Various Age Groups in Different Quartiles of the INFLA-Score.

Q1 = The Lowest INFLA-score quartile; Q2 = The Second-lowest INFLA-score quartile; Q3 = The Third-lowest INFLA-score quartile; Q4 = The Highest INFLA-score quartile.

Supplementary Figure 9. The Associations Between Different Dietary Pattern Scores and Age-related Hospitalization and Premature Death.

Values are n/N (case number/total number) or HR (95% CI). Results were adjusted for age, sex, race, smoking, drinking, BMI, MET, Townsend deprivation index, hypertension, mineral supplements intake, vitamin supplements intake, aspirin and other NSAIDs intake, medicines for hypercholesterolemia, hypertension and diabetes intake; CVD, Cancer, Diabetes, and Respiratory disease at baseline.

BMI = Body Mass index; MET = metabolic equivalent; NSAIDs = Non-Steroidal Anti-Inflammatory Drugs; CVD = cardiovascular disease; HEI-2020 = Healthy Eating Index-2020, MED = Mediterranean, DASH = Dietary Approaches to Stop Hypertension; T = tertile.
